# The Effect of Ramadan Fasting and Physical Activity on Body Composition, Serum Osmolarity Levels and Some Parameters of Electrolytes in Females

**DOI:** 10.5812/ijem.9602

**Published:** 2013-04-01

**Authors:** Seyyed Reza Attarzadeh Hosseini, Mohammad Ali Sardar, Keyvan Hejazi, Samaneh Farahati

**Affiliations:** 1 Sport Physiology Faculty of Physical Education and Sport Sciences, Ferdowsi University of Mashhad, Mashhad, IR Iran; 2 Department of General Courses, Faculty of Medicine, Mashhad University of Medical Sciences, Mashhad, IR Iran; 3 Department of Physical Education and Sports Sciences, Ferdowsi University of Mashhad, Mashhad, IR Iran

**Keywords:** Fasting, Osmolar Concentration, Serum

## Abstract

**Background:**

So far, there have been a few and incoherent results about the effects of physical activities. Fasting in Ramadan has an effect on the level of osmolarity and the concentration of serum electrolytes both in active and inactive females.

**Objectives:**

The aim of this study was to observe the changes of serum electrolytes and osmolarity levels according to regular exercise during fasting.

**Patients and Methods:**

Twenty two healthy females who were elected by convenience sampling method were divided into two groups: 1) fasting + exercise (FE; n = 11) and 2) fasting + non exercise (FNE; n = 15). The FE group participated in aerobic training for four sessions per week during the fasting. All measurements were done once before the first day, on the second week, on the fourth week and two weeks after fasting month and these measures were used to analyze test results.

**Results:**

The mean differences were as follows: significant weight loss, BMI, WHR, in two groups at the end of Ramadan (P < 0.05). The mean of weight, BMI, WHR, body fat, protein, mineral and total water showed no difference between groups (P > 0.05). Potassium, creatinine, urea and uric acid had been decreased significantly in both groups (P < 0.05). Variance between groups was significant only in variable urea (P < 0.05). Variations within group had been changed in FBS; sodium, phosphorus and osmolarity levels were not changed significantly.

**Conclusions:**

According to this result, regular exercise in case of fasting in Ramadan led to some changes in serum osmolarity index, electrolytes and water. Therefore, it is important for female athletes to consider applying a suitable nutritious diet and sufficient water consumption during Ramadan

## 1. Background

Ramadan is the most appreciated lunar month among Muslims which restricts eating, drinking, and smoking (1). There are some changes in eating habits, receiving energy, sleeping and daily physical activities due to the properties of this monthwhich may also lead to physiological changes and hematological and biochemical changes of blood (2, 3). Research indicated that fasting in Ramadan can cause changes in body components, serum electrolyte intensity, and osmolarity (4). Likewise, Salehi et al. (5) noticed a significantdecrease in body weight, body mass index, glucose, and serum cholesterol after a complete period of fasting in Ramadan, while, Saada et al. (6) proved that there is not a significant change in body mass index between fasters and non-fasters in Ramadan while glucose, HDL-C, urea, creatinine, and protein increased significantly, and hemoglobin and total cholesterol levels decreased within three weeks. There are various results about the effects of fasting on electrolyte and osmolarity. Indral et al. (7) found a significant reduction in serum urea, triglycerides, total cholesterol, and LDL-C amounts in 19 blood samples of fasting men during the first to 23rd day of Ramadan. Azwany et al. (8) examined the effects of one month of fasting on 43 Muslims. They reported a significant increase in urinary osmolarity after four weeks of fasting regarding the fact that the amount of water absorption was natural. After 4 weeks, there was no significant change in blood urea. This happened while Nomani et al. (9), had reported a significant increase in blood urea level by the end of Ramadan. In a long study, Azizi (10) stated that during long-term hunger pangs, uric acid of serum increases abnormally, which is thought to be caused by Glomeruli filtration (GRF) and uric acid release. However, In Islam the uric acid level increases slightly which is caused by short-term, long-term, and variable fasting. Moreover, Azizi (11) mentioned that fasting in Ramadan does not bring about a considerable change in sodium and potassium serums, After studying the effects of fasting on potassium change in 10 young and healthy men between 18 to 25. Morilla et al. (12) concluded that potassium is reduced mainly in the mornings and its amount increases in the afternoons; in addition, its discharge rate only increases within the fourth week. Azizi (10) reported that long-term fasting will cause an abnormal increase in uric acid amounts, stability in osmolarity of urinary volume, Ph, nitrogen, discharged electrolytes, and sodium. However, the evidence suggests that doing physical activities, during Ramadan, will facilitate body metabolism, and will also prevent fat accumulation in body; on the other hand, it has also been reported that fasting athletes face fewer digestive and blood sugar problems (13); but there are few studies about the effects of Ramadan Fasting along with regular exercises on the level of serum osmolarity and electrolytes concentration. Ronald et al. (14) pointed out that fasting along with physical activity will lead to more energy consumption, perspiration, and dehydration which can cause mild or strong malnutrition and liquid and electrolyte imbalance in the body. The controversial results about the effects of fasting on serum osmolarity level, parameters of electrolytes, body composition in conducted studies along with the lack of sufficient evidence in examining the effects of physical activities in the holy month of Ramadan and the increase in fasting time in the hot season of summer have validated the examination of fasting with or without regular physical activities.

## 2. Objectives 

The aim of the present study was to investigate the changes in body composition, serum osmolarity levels and some parameters of electrolytes responses to moderately aerobic physical performance during Ramadan fasting.

## 3. Patients and Methods

## 3.1. Subjects

Twenty six healthy overweight women aged 20-45 years old (body mass index above 25 kg/m2), who were employees of the Ferdowsi University of Mashhad, volunteered to participate in this study. During the first stage, all volunteers were asked to complete a medical test as well as a medical questionnaire to make certain that they were not taking any regular medications, were free from cardiac, renal, metabolic diseases, and respiratory (15). Also, all volunteers were completely familiar with all of the experimental procedures and exercise protocol. The ethical comitte of Mashhad University of Medical Sciences has approved the studey. They were also made clear about their right to withdraw anytime from the study if they wished so. The volunteers were classified randomly into two experimental groups: 1) Fasting + Exercise (FE; n = 15) and 2) Fasting + non-exercise (FNE; n = 11). The following equation was used to determine sample size:

## 3.2. Study Design

This research was semi-experimental with two experimental groups. The study was conducted in Mashhad in 2012 when Ramadan fasting occurred between July 21 and August 18 (Hijri year 1433). The weather at the time of training was warm: ambient temperature was approximately 30-40°C with a relative humidity of 25-30%. Experiments were performed in four separate sessions: 1) first week before Ramadan (Pre-R) which represented the baseline session; 2) during the second week of Ramadan (Med-R); 3) during the fourth week of Ramadan (End-R); 4) two weeks after the end of Ramadan month (After-R). All tests were performed at 14.00-15.00 in the same order. All subjects had almost the same life style, sleep habits and food intake schedule.

## 3.3. Anthropometric Measurements

The body mass index (BMI) was measured by height and weight values as follows: weight (kg)/height (m^2^) and waist-to-hip ratio (WHR). The body composition was determined by bioelectric impedance using In Body-720 (Biospace, Dogok-dong, South Korea) to study the fat mass (FM), muscle mass (MM) and total body water (TBW). The analysis performed each time under standardized conditions; 4 hour fasting and not having intense physical exercise 12 hours before the test and in fourth week of Ramadan, before midday, any menstrual cycle and ± 2 days. The height of the same person was measured by using an electronic balance with stadiometer (SECA-Germany) to the nearest 0.1 cm (16).

## 3.4. Exercise Programs

The women training program began the morning of the first day of 30th of Ramadan (11 to 12). It included 4 sessions/week, 60 min/session. The exercise protocol included: 10 min general warm up (walking, stretching and movement exercise); 45 min aerobic training like Jogging and aerobic exercise with intensity of 50-65 percent of maximum heart rate reserve (MHRR. At the end of each exercise session, activities like jogging, walking and stretching were done for 5 minutes to return the body to its normal condition (17). According to the MHRR, every single athlete was respectively measured based on Karvonen equation (1) and was also controlled during exercise by a heart rate monitor (made in Finland–Polar)(18).

Equation (1):

Target heart rate= (%60 or %70+ (((220- age)- Resting heart rate)) + Resting heart rate

Heart rate monitors (Polar Team Sports System, Polar Electro, Finland) were then fitted, before preparation and application of the sweat patches. Volunteers then used their training kit, switched on their heart rate monitors, and stood quietly for approximately one minute for measurement of resting heart rate before proceeding to the field of training. Immediately after training, the volunteers switched off and detached their heart rate monitor before having their sweat patches removed and being re-weighed.

## 3.5. Biochemical Tests

For examining the parameters of this study, 5 ml of venous blood obtained at four occasions from each subject: the first session took place one week before Ramadan; the second session in the second week of Ramadan; the third session in the fourth week of Ramadan, and the fourth session two weeks after Ramadan. The time of blood sampling in the study was 9-10 a.m., at which all volunteers were fast. Participants were asked to avoid eating for 24 hours before intense physical activities like cycling, running and walking which last more than 15 minutes. Blood samples in all related studies were collected by venepunction from forearm vein after at least 15 minutes of resting or in the supine position. Fast blood sugar was determined by flame photometry (glucose oxidase method; coefficient of variation (CV), CV = 1.97%) using auto analyzer unit (CRONIX 801). For measurement of serum electrolytes, such as sodium, potassium, calcium, phosphate, creatinine, urea and uric acid, standard methods were used are as follow: Sodium and potassium were determined by flame photometry method using flame photometer unit (GDV; co-production in Italy and the U.S.); also, calcium, phosphate, creatinine, urea and uric acid were determined by flame photometry using auto analyzer unit (CRONIX 801). Calcium was determined by Arsenazo method with coefficient of variation (CV), CV = 1.03%; Phosphorus was determined by the Ammonium molybdate With CV = 1.14%; Creatinine was measured by Jaffe kinetic method. With CV = 0.98%; Urea was measured by the urease method. With CV = 1.21% and Uric acid was measured by the uricase method with CV = 1.54%. The quality control tests were done based on the laws of VSTGARD by drawing Levy Jenning quality control and testing on control serum Trulab N. Moreover, serum osmolarity index was calculated using a linear equation 2 (19).

Linear equation (2):

Serum osmolarity = (Na× 2) + (Glucose/18) + (Urea/2.8)

*3.6.* *Statistical Analysis*

Descriptive statistics (Mean ± SD) were calculated for all variables. Statistical analyses of the data were carried out using ANOVA for repeated measures of two groups (exercise with fasting and non-exercise with fasting). Post-hoc testing was accomplished using Bonferroni. Differences were considered significant (P < 0.05). The data were analyzed using SPSS (SPSS Inc., Chicago, IL) version 16.

## 4. Results 

According to (Table 1), our results show a significant weight loss, BMI, WHR in two groups by the end of Ramadan (P < 0.05). Results of the effect of Ramadan fasting on serum osmolarity level and some parameters of electrolytes show an increase during Ramadan (Table 2). Potassium, creatinine, urea, uric acid decreased significantly in both groups (P < 0.05). Difference between groups was significant only in urea level (P < 0.05). Differences within group in FBS, sodium, phosphorus and osmolarity levels did not change significantly (P>0.05). Variation in variance between groups such as: FBS, sodium, phosphorus and osmolarity levels was not significant (P > 0.05).

**Table 1.** Changes in Body Size and Body Composition of the FE (n = 15) and FNE (n = 11) During Different Stages and Changes in Means of Within and Between Groups

Variables	Groups	Stages		Variations			
		Week before Ramadan, Mean ± SD	Week after Ramadan	P Value ^ a^		P Value ^ b^	
				F	P	F	P
**Weight, Kg**	FE^ c^	79.9 ± 13.2	78.4 ± 13.2	25.358	0.001	2.899	0.105
	**FNE**	71.1 ± 9.6	69.9 ± 9.0				
**BMI, Kg/m^2^**	FE	32.0 ± 4.4	31.4 ± 4.7	25.041	0.001	3.786	0.067
	**FNE**	28.6 ± 3.2	28.1 ± 2.9				
**WHR, Cm**	FE	0.94 ± 0.06	0.92 ± 0.07	8.275	0.010	1.005	0.329
	**FNE**	0.91 ± 0.06	0.90 ± 0.06				
**Body fat Percent, %**	FE	44.4 ± 5.7	43.9 ± 5.2	0.526	0.477	1.328	0.264
	**FNE**	41.6 ± 4.4	41.7 ± 4.5				
**Minerals, Kg**	FE	3.00 ± 0.48	2.99 ± 0.47	1.412	0.249	0.676	0.421
	**FNE**	2.87 ± 0.31	2.83 ± 0.29				
**Protein, g/dL**	FE	8.60 ± 1.2	8.53 ± 1.2	2.770	0.112	0.418	0.248
	**FNE**	8.09 ± 0.93	7.95 ± 0.83				
**Water, Kg**	FE	32.3 ± 4.5	32.0 ± 4.4	3.509	0.077	0.544	0.229
	**FNE**	30.3 ± 3.4	29.7 ± 3.0				

^a^P Value within group

^b^P Value between group

^c^Abbreviations: FE, fasting + exercise; FNE, fasting + non-exercise

**Table 2.** Changes in Serum Electrolyte Values in the FE (n = 15) and FNE (n = 11) During Different Stages and Changes in Means of Within and Between Groups

Variables	Groups	Stages				Variations			
		Week Before Ramadan Mean ± SD	Day 15 of Ramadan Mean ± SD	Day 28 of Ramadan Mean ± SD	Week After Ramadan, Mean ± SD	P Value ^ a^		P Value ^ b^	
						F	P	F	P
**FBS, mg/** **dL**	FE	82.2 ± 5.5	84.4 ± 5.6	81.4 ± 6.1	83.3 ± 6.1	1.03	0.384	3.592	0.073
	**FNE**	87.2 ± 5.1	86.3 ± 7.2	83.3 ± 7.9	86.6 ± 6.0				
**Sodium, Meq/ liter**	FE	139.7 ± 1.9	140.4 ± 1.2	140.0±1.7	139.7±1.7	0.781	0.509	0.813	378
	**FNE**	139.0±1.6	139.6±1.2	139.3±1.8	140.4±1.5				
**Potassium,(Meq/liter)**	FE	4.0±0.25	4.1±0.27	4.0±0.23	4.0±0.24	3.29	0.026	0.109	0.745
	**FNE**	4.2±0.15	4.1±0.25	3.9±0.23	3.9±0.16				
**Calcium, (Meq / liter)**	FE^c^	9.1±0.28	9.1±0.38	9.0±0.27	9.2±0.32	0.273	0.844	0.632	0.236
	**FNE**	9.1±0.38	9.1±0.41	9.1±0.45	9.1±0.20				
**Phosphorus, mg/dL**	FE	3.6 ± 0.35	3.5 ± 0.37	3.6 ± 0.19	3.6 ± 0.45	1.163	0.332	0.171	0.684
	**FNE**	3.7 ± 0.92	3.4 ± 0.62	3.5 ± 0.52	3.5 ± 0.47				
**Creatinine(mg/dL)**	FE	0.78 ± 0.05	0.83 ± 0.07	0.82 ± 0.05	0.82 ± 0.07	3.618	0.038	0.622	0.440
	**FNE**	0.77±0.06	0.81±0.10	0.76±0.07	0.83±0.05				
**Urea, mg/dL**	FE	27.3 ± 6.6	27.0 ± 6.9	26.6 ± 5.8	28.0 ± 7.3	3.682	0.017	4.857	0.039
	**FNE**	22.3 ± 3.8	24.4 ± 3.7	19.5 ± 3.6	23.4 ± 2.4				
**Uric acid, mg/dL**	FE	5.0 ± 0.86	4.6 ± 0.97	5.2 ± 0.88	4.8 ± 0.74	5.027	0.004	0.001	0.979
	**FNE**	5.2 ± 0.91	4.6 ± 0.61	5.0 ± 0.99	4.7 ± 0.32				
**Osmolarity, mg/dL**	FE	293.8 ± 5.5	294.3 ± 3.9	294.2 ± 4.6	294.1 ± 4.7	1.89	0.140	2.616	0.121
	**FNE**	290.8 ± 1.9	292.8 ± 1.7	290.2 ± 3.7	294.0 ± 3.1				

^a^P Value within group

^b^P Value between group

^c^Abbreviations: FE, fasting + exercise; FNE, fasting + non-exercise

## 5. Discussion 

Fasting in the holy month of Ramadan is a popular practice amongst Muslims. It is the abstinence from eating, drinking and smoking from about an hour before the sunrise until sunset (20).

According to these results from weight, BMI and WHR in two groups decreased significantly till the end of Ramadan. However, body fat percentage of two groups decreased slightly.

In other words, during Ramadan, active females underwent a significant reduction in body mass index, waist to hip ratio and body fat percentage compared to inactive females. Generally, body weight is one of the items which researchers anticipated to be changed in Ramadan; however, based on the conducted research, body weight change during Ramadan is variable. Studies show that there are conflicting results on changes in body weight during Ramadan (11, 21). Some studies show no change in weight (22, 23), while others show an increase instead of decrease (24). There is controversy in the results obtained by Ziaee et al. (25); Fakhrzadeh et al. (25); Al-Hourani et al. (26) and Trabelsi et al. (27) showed a decrease in body mass and fat mass during Ramadan; while Yucel et al. (28) showed no change; moreover Frost and Meckel et al. (29); Gharbi et al. (30) reported an increase in weight , fat gain and energy consumption. Hence, Fakhrzadeh et al. (31) reported that fasting caused a significant reduction in weight and BMI in men, and in waist circumference in women. Al-Hourani et al. (26) reported that the body weight and BMI decreased significantly during Ramadan fasting. According to the results of numerous studies, Loss of weight, body mass index, waist-to-hip circumference ratio and body water was confirmed, While, the findings of the present study are compatible the results of Yucel et al. (28), Meckel et al. (29) and Gharbi et al. (30). It has to be mentioned that other elements such as metabolic changes resulted from different diets, consumed food types, activity level, climate, and the reduction in participants’ metabolistic rest-time are all other elements of influence (32). In addition to that, Ramadan was in the summer; therefore, our research results might be different compared to those gained in the autumn since fasting periods are different. Many previous studies which have been published about the effect of fasting on renal function tests-blood urea, serum createnine and albumin- in healthy individuals reported small changes that were not statistically significant (2, 33). In this study, potassium levels decreased, while creatinine, urea and uric acid increased significantly in both groups. Variance between groups only in variable urea was significant. Studies report that the effect of Ramadan fasting on osmolarity levels has been conflicting and inconsistent. This finding was supported by Mohammed (34), Miladipour et al. (35). However, the findings of the present study were inconsistent with Sadiya et al. (36), Boobes et al. (37), U¨ nalacak et al. (38) and Bernieh et al. (39). Mohammed (34) reported that no significant changes were observed in blood urea and serum albumin levels. Whereas, serum uric acid and total serum protein levels were statistically lower during and after Ramadan. Miladipour et al. (35) reported that total excretion of calcium, phosphate, and magnesium in 24-hour urine and also urine volume during fasting were significantly lower than those in the non-fasting period. Furthermore, urine concentration of calcium during fasting was significantly lower than those of non-fasting period. Sadiya et al. (36) found that kidney function, plasma Creatinine, and urea concentrations remained unchanged. Boobes et al. (37) showed that fasting caused no significant change in urea acid, sodium, potassium, urea, creatinine and albumin during the month of Ramadan. Bernieh et al. (39) indicated that there was no significant change in serum and urine. Biochemical analysis showed no significant changes. Also, there was a significant elevation of serum potassium levels. The increase in blood urea intensity might be due to an increase in protein catabolism, doing physical activity, or the reduction of blood circulation in kidneys. Some studies reported that the increase of blood urea, which may be due to some physical activities, in fact, stimulates energy consumption and causes reduction in gained energy (40). Similarly, when an individual is under physical stress, albumin and urea discharge increases in the body. Physical exercise is one of the elements which can change biochemical factors. Protein increases in the daily diet. Intestine-stomach bleeding, body dehydration, and inadequate liquid consumption are some of the other elements which can increase blood urea level in fasting periods (40). In this study, creatinine levels were increased significantly at the end of the Ramadan. Creatinine, which is a dischargeable substance, is mainly produced in the muscles and is a good criterion to check kidneys’ health since if it is not removed from blood by kidneys, its plasma intensity increases. Sometimes, long-term fasting, thirst, and dehydration will gradually increase the amount of creatinine in the body, which its intensity decreases if dehydration is over (41). During continual physical exercise, especially in Ramadan, conserving body liquid resources and balancing outer-cell liquid with inter-tissue spaces is crucial. Osmolarity, which is the sign of osmosis activity of all plasma particles, is between 280-295 mili-osmol in one kilogram in mature people. Despite the fact that in our research, osmolarity changes, , in both active and inactive fasting groups, were considered normal, the results indicated that intra-group average changes of osmolarity serum in the active fasting group increased but not significantly. The sensitivity level of Osmosis receptors is about one percent that is due to changes to Osmolarity which can cause the discharge of anti-diuretic hormones and stimulating thirst. Therefore, body liquid change and the amount of outer-cell sodium can make a lot of changes in Osmolarity (42). Osmolarity increase might be due to dehydration and not consuming enough liquids during the day. In long-term fasting, kidneys re-absorb water. With respect to the fact that sodium levels of both active and inactive fasting groups were not changed, and also the fact that osmolarity level is mainly affected by outer-cell sodium, low amounts of osmolarity is justifiable. The limitations of our study included variety of diets, different adaptation responses to fasting and individual differences of female participants.

## References

[1] Elnasri H, Ahmed A (2006). Effects of Ramadan fasting on blood levels of glucose, triglyceride and cholesterol among type II diabetic patients.. Sudanese J Public Health..

[2] Aksungar FB, Eren A, Ure S, Teskin O, Ates G (2005). Effects of intermittent fasting on serum lipid levels, coagulation status and plasma homocysteine levels.. Ann Nutr Metab..

[3] Tayebi S, Hanachi P, Niaki A (2010). Ramadan fasting and weight-lifting training on vascular volumes and hematological profiles in young male weight-lifters.. Global J health..

[4] Furuncuoglu Y, Karaca E, Aras S, Yönem A (2007). Metabolic, Biochemical and Psychiatric Alterations in Healthy Subjects During Ramadan.. Pakistan J Nutrition..

[5] Salehi M, Neghab M (2007). Effects of fasting and a medium calorie balanced diet during the holy month Ramadan on weight, BMI and some blood parameters of overweight males.. Pak J Biol Sci..

[6] Saada A, Selselet attou G, Belkacemi L, Ait chabane O, Italhi M, Bekada AM (2010). Effect of Ramadan fasting on glucose, glycosylated haemoglobin, insulin, lipids and proteinous concentrations in women with non-insulin dependent diabetes mellitus.. African J Biotech..

[7] Indral M, Satumanl L, Widodo E, Tinny E, Endang S, Soemardini S (2007). Study of some biochemical parameters in young men as effected by Ramadan Fasting.. J Kedokteran Yarsi..

[8] Azwany N, Aziz A, Mohammad W (2004). The Impact of ramadan fasting on hydration status of type 2 diabetics in kubang kerian, kelantan.. J Kesihatan Masyarakat Zsu Khas..

[9] Nomani M (1997). Dietary Fat, Blood cholesterol and Uric Acid Levels during Ramadan Fasting.. Int J Ramadan Fasting Res..

[10] Azizi F (2002). Research in Islamic fasting and health.. Ann Saudi Med..

[11] Azizi F, Rasouli H (1987). Serum glucose, bilirubin, calcium, phosphorus, protein and albumin concentrations during Ramadan.. Med J IR Iran..

[12] Morilla R, Rodrigo J, Caravaca A, Villaverde G, Pérez B, Moreno Y (2011). Changes of the potassium ion during the fast of Ramadan. preliminary outcomes.. Nutr Hosp..

[13] Zerguini Y, Dvorak J, Maughan RJ, Leiper JB, Bartagi Z, Kirkendall DT (2008). Influence of Ramadan fasting on physiological and performance variables in football players: summary of the F-MARC 2006 Ramadan fasting study.. J Sports Sci..

[14] Maughan RJ, Bartagi Z, Dvorak J, Zerguini Y (2008). Dietary intake and body composition of football players during the holy month of Ramadan.. J Sports Sci..

[15] Shephard R (1991). Readiness for physical activity.. Sports Med..

[16] National Health and Nutrition Examination Survey (NHANES) (2004). Anthropometry Procedures Manual..

[17] Pollock ML, Wilmore J (1990). Exercise in health and disease: Evaluation and prescription for prevention and rehabilitation..

[18] Robbert A, Landwehr R (2002). The supporting history of the "HRmax=220-age" equation.. J Exercise Physiol online..

[19] Palmer B, Sterns R (2009). Fluid, electrolytes, and acid-base disturbances..

[20] Sarraf-Zadegan N, Atashi M, Naderi GA, Baghai AM, Asgary S, Fatehifar MR (2000). The effect of fasting in Ramadan on the values and interrelations between biochemical, coagulation and hematological factors.. Ann Saudi Med..

[21] Mafauzy M, Mohammed WB, Anum MY, Zulkifli A, Ruhani AH (1990). A study of the fasting diabetic patients during the month of Ramadan.. Med J Malaysia..

[22] Laajam MA (1990). Ramadan fasting and non-insulin-dependent diabetes: effect on metabolic control.. East Afr Med J..

[23] Sulimani RA, Laajam MA, Al-Attas O, Famuyiwa FO, Bashi S, Mekki MO (1991). The effect of ramadan fasting on diabetes control in type II diabetic patients.. Nutrition Res..

[24] Rashed AH (1992). The fast of Ramadan.. BMJ..

[25] Ziaee V, Razaei M, Ahmadinejad Z, Shaikh H, Yousefi R, Yarmohammadi L (2006). The changes of metabolic profile and weight during Ramadan fasting.. Singapore Med J..

[26] Al-Hourani HM, Atoum MF (2007). Body composition, nutrient intake and physical activity patterns in young women during Ramadan.. Singapore Med J..

[27] Trabelsi K, El Abed K, Trepanowski JF, Stannard SR, Ghlissi Z, Ghozzi H (2011). Effects of ramadan fasting on biochemical and anthropometric parameters in physically active men.. Asian J Sports Med..

[28] Yucel A, Degirmenci B, Acar M, Albayrak R, Haktanir A (2004). The effect of fasting month of Ramadan on the abdominal fat distribution: assessment by computed tomography.. Tohoku J Exp Med..

[29] Meckel Y, Ismaeel A, Eliakim A (2008). The effect of the Ramadan fast on physical performance and dietary habits in adolescent soccer players.. Eur J Appl Physiol..

[30] Gharbi M, Akrout M, Zouari B (2003). [Food intake during and outside Ramadan].. East Mediterr Health J..

[31] Fakhrzadeh H, Larijani B, Sanjari M, Baradar-Jalili R, Amini MR (2003). Effect of Ramadan fasting on clinical and biochemical parameters in healthy adults.. Ann Saudi Med..

[32] Sweileh N, Schnitzler A, Hunter GR, Davis B (1992). Body composition and energy metabolism in resting and exercising muslims during Ramadan fast.. J Sports Med Phys Fitness..

[33] Yousef B, Bassam B, Raafat A (2009). Fasting Ramadan in kidney transplant is safe.. Saudi J kidney Dis Transpl..

[34] Mohammed Z (2011). The Influence of Ramadan fasting on some hematological and biochemical parameters in healthy adult males.. Iraqi National J for Nursing Specialties..

[35] Miladipour AH, Shakhssalim N, Parvin M, Azadvari M (2012). Effect of Ramadan fasting on urinary risk factors for calculus formation.. Iran J Kidney Dis..

[36] Sadiya A, Ahmed S, Siddieg HH, Babas IJ, Carlsson M (2011). Effect of Ramadan fasting on metabolic markers, body composition, and dietary intake in Emiratis of Ajman (UAE) with metabolic syndrome.. Diabetes Metab Syndr Obes..

[37] Boobes Y, Bernieh B, Al Hakim MR (2009). Fasting Ramadan in kidney transplant patients is safe.. Saudi J kidney Dis Transpl..

[38] Unalacak M, Kara IH, Baltaci D, Erdem O, Bucaktepe PG (2011). Effects of Ramadan fasting on biochemical and hematological parameters and cytokines in healthy and obese individuals.. Metab Syndr Relat Disord..

[39] Bernieh BO, Mohamed AO, Wafa AM (1994). Ramadan fasting and renal transplant recipients: Clinical and biochemical effects.. Saudi J kidney Dis Transpl..

[40] Degoutte F, Jouanel P, Begue RJ, Colombier M, Lac G, Pequignot JM (2006). Food restriction, performance, biochemical, psychological, and endocrine changes in judo athletes.. Int J Sports Med..

[41] Haghdoost AA, Poorranjbar M (2009). The interaction between physical activity and fasting on the serum lipid profile during Ramadan.. Singapore Med J..

[42] Grimshaw P (2006). Sport and exercise biomechanics..

